# Optimising Psychosocial Interventions for Parents Following Perinatal Bereavement: A Qualitative Study of Midwives' Perspectives

**DOI:** 10.1111/jan.70334

**Published:** 2025-11-03

**Authors:** Jiaying Xie, Annmarie Grealish, Linda Biesty, Andrew Hunter

**Affiliations:** ^1^ School of Nursing and Midwifery University of Galway Galway Ireland; ^2^ School of Nursing and Midwifery Health Research Institute, University of Limerick Limerick Ireland; ^3^ Florence Nightingale Faculty of Nursing, Midwifery & Palliative Care King's College London London UK

**Keywords:** induced abortion, midwifery, nurse midwives, parents, perinatal bereavement care, perinatal death, perinatal loss, psychosocial intervention, spontaneous abortion, stillbirth

## Abstract

**Aim:**

To explore midwives' experiences of providing psychosocial interventions to parents following perinatal bereavement in maternity care settings.

**Design:**

A descriptive qualitative study.

**Methods:**

Twenty‐two midwives were recruited from three maternity services in Ireland using purposive and snowball sampling. Semi‐structured interviews were conducted between July and November 2024. Reflexive thematic analysis was used to analyse the data.

**Results:**

Four themes were identified: (1) Building relationships as a foundation for psychosocial intervention delivery; (2) Psychosocial intervention as the core element of perinatal bereavement care; (3) Negotiating intervention delivery in a constrained system; and (4) Navigating emotional labour and professional growth. Midwives advocated provision of compassionate and relationship‐based psychosocial interventions, but often faced systemic barriers, limited guidance, and insufficient training. Supportive structures and psychosocial intervention focused training were seen as critical to sustaining care quality and midwives' wellbeing.

**Conclusion:**

Applying the Socio‐Ecological Model (SEM) revealed that midwives' delivery of PSIs to support grieving parents after perinatal bereavement is influenced by multi‐level factors, underscoring the need for policy integration, institutional support, and contextually grounded, midwife‐led approaches.

**Implications for the Profession and/or Patient Care:**

Integrating SEM into intervention design can guide the development of multi‐component PSIs that address multilevel influences and align with both parents' needs and midwives' capacities.

**Impact:**

This adds to the understanding of how midwives deliver psychosocial interventions in perinatal bereavement care. Midwives view the delivery of these interventions as central to their role, while acknowledging the need for the development of, and training in structured, midwife‐led psychosocial interventions in perinatal bereavement care.

**Reporting Method:**

COREQ.

**Patient or Public Contribution:**

Patients and members of the public were involved in study design, data collection and validation of findings. Their contributions included reviewing protocols and recruiting materials, facilitating recruitment and participating in advisory groups, ensuring the relevance and sensitivity of the research.

**Summary**

- What does this paper contribute to the wider global clinical community?
  ○ Offers novel insights into midwives' experiences of delivering psychosocial interventions for bereaved parents in real world maternity care settings.
  ○ Identifies critical gaps in systemic support, training, and policy that limit consistent bereavement care.
  ○ Provides evidence to guide the development of structured, midwife‐led psychosocial interventions.

## 1 Introduction

The World Health Organization (WHO 2006) defines perinatal death as the death of a fetus or neonate from 22 completed weeks of gestation to seven completed days after birth. However, in academic research, this concept is often expanded to include pregnancy loss from conception up to 28 days after birth, encompassing ectopic pregnancy, miscarriage, termination of pregnancy, stillbirth, neonatal death (Fenstermacher and Hupcey 2013; Fernandez‐Basanta et al. 2023; HSE 2022; Shaohua and Shorey 2021). Perinatal bereavement, which commences immediately following the loss of a pregnancy, is characterised by a complex emotional response, most notably grief, and can have significant and long‐lasting impacts on the emotional wellbeing of parents (Dolan et al. 2022; Fenstermacher and Hupcey 2013). In these critical moments, the compassionate support provided by healthcare professionals plays a vital role in shaping parents' grieving process (Ravaldi et al. 2018). Midwives may be the first and most consistent point of contact for parents during and after pregnancy loss (Dolan et al. 2022; Xie et al. 2024). Compassionate emotional support and clear, empathetic communication from midwives have been shown to significantly influence parents' psychological adjustment and promote healthier long‐term emotional outcomes (Berry et al. 2021; McNamara et al. 2022; Xie et al. 2024). Given this central role, it is essential to understand how midwives provide emotional and psychosocial support in the context of perinatal bereavement.

## 2 Background

Psychosocial interventions (PSIs) are broadly defined as non‐pharmacological approaches aimed at addressing psychological, social, and relational distress, particularly in individuals experiencing mental health challenges (Barbui et al. 2020). PSIs are frequently implemented in settings with overwhelming needs and few resources and are often delivered by non‐specialist health professionals, including nurses without mental health training, lay health workers, or peer support workers (Purgato et al. 2018). In the context of perinatal bereavement, PSIs are intended to alleviate parents' psychological distress and support parents as they navigate grief and loss (Charrois et al. 2020). Emerging evidence suggests that PSIs, such as bereavement counselling, expressive writing, peer support, family‐based programs, psychoeducation and psychotherapy, can reduce emotional distress, grief, depression, anxiety and post‐traumatic stress among parents following perinatal bereavement (Shaohua and Shorey 2021; Xie, Hunter, et al. 2022; Xie, Tang, et al. 2022).

Early and positive contact with attentive health professionals has been shown to facilitate the mourning process for bereaved parents, helping to address their emotional needs and promote recovery (Kingdon et al. 2015; Malm et al. 2011; Martinez‐Serrano et al. 2018). Midwives, in particular, are well placed to provide compassionate support for bereaved parents during perinatal loss, as they may be the first and primary point of contact and are continuously involved in providing bereavement care throughout maternity hospital care (Homer et al. 2016). In Ireland, the National Standards for Bereavement Care Following Pregnancy Loss and Perinatal Death published by the Health Service Executive (HSE 2022) position midwives as central to perinatal bereavement care, with responsibilities for emotional support, memory‐making, and linking families to multidisciplinary and community resources. Specialist roles such as the Clinical Midwife Specialist (CMS) in bereavement further enhance the continuity and quality of care through advanced expertise, coordinated support, and staff education (HSE 2022). Within Irish maternity care, midwives are thus identified as the key providers of PSIs to bereaved parents during hospital care.

Our recent review (Xie, Hunter, et al. 2022; Xie, Tang, et al. 2022) illustrates how midwife‐led PSIs can improve symptoms of grief, anxiety, depression, posttraumatic stress disorder and better psychosocial outcomes for parents following perinatal loss. However, this review also revealed that midwives often feel underprepared, which can result in inconsistent quality of care, feelings of inadequacy, emotional distress, and burnout (Ravaldi et al. 2022, 2018). Systemic barriers, such as workload pressures, staffing shortages, lack of continuity of care, and limited access to specialist services, further hinder their ability to provide consistent and compassionate support (Garcia‐Catena et al. 2023). Meanwhile, despite the introduction of national standards in 2016 and their update in 2022, the 2023 review still identified significant gaps in mental health support, communication, and continuity of care for bereaved parents. This ongoing disconnect between policy and practice underscores the importance of understanding how midwives engage with PSIs, including the strategies they employ and the challenges they encounter, in order to bridge this gap and ensure parents receive meaningful, consistent support. Nevertheless, evidence exploring midwives' experiences of delivering PSIs within clinical settings remains limited (Xie et al. 2024).

To address this gap, the present study explores midwives' experiences of providing PSIs to parents following perinatal bereavement in the Irish maternity care context, with particular focus on the current practices, barriers, enablers, and support and training needs. Moreover, insights from this study can inform the development of a structured PSI framework, targeted training programs, and systemic improvements in maternity care to enhance the delivery of PSIs. Accordingly, the research question guiding this study is: what are the experiences of midwives in providing PSIs to parents experiencing perinatal bereavement in the Irish maternity care setting?

## 3 The Study

The aim of the study was to explore midwives' perceptions and experiences of providing PSIs to support parents through the grieving process following perinatal bereavement in the Irish maternity care setting.

## 4 Methods

### 4.1 Design

A descriptive qualitative design (Sandelowski 2010, 2000) was employed to explore midwives' experiences of providing PSIs in perinatal bereavement care within the context of Irish maternity care. Descriptive qualitative research is suited to capturing both the factual elements of participants' accounts and the meanings they assign to their experiences, enabling the identification of common patterns to inform future intervention development (Sandelowski 2010, 2000; Willis et al. 2016). This approach also allowed for a rich, context‐sensitive understanding of how midwives make sense of PSI in practice (Willis et al. 2016). The Consolidated Criteria for Reporting Qualitative Research (COREQ) checklist (Data S1) was followed to enhance the transparency of this study's reporting (Tong et al. 2007).

#### 4.1.1 Patient and Public Involvement (PPI)

A steering group was established at the outset of the study, comprising a clinical midwife, a CMS in bereavement, and three women with lived experience of perinatal bereavement, to enhance the study's quality and relevance to participants. Through meetings and email correspondence, the group reviewed the study protocol, advised on recruitment materials and interview questions, and provided feedback on the thematic findings. Their input informed refinements to the study design and improved the sensitivity of language, helping ensure the research was responsive to participants' needs. The Guidance for Reporting Involvement of Patients and the Public (GRIPP2) reporting checklist (Staniszewska et al. 2017) was used to guide the reporting of PPI contributions and impact on this study (Data S2).

### 4.2 Study Setting and Recruitment

This study was conducted in three publicly funded maternity sites in Ireland to capture varied service contexts (DoH 2016): Site 1 is a high‐volume tertiary hospital (> 8000 births/year, urban), Site 2 is a standalone regional hospital (3000–8000 births/year), and Site 3 is a regional unit within a general hospital (< 3000 births/year). All operate under the HSE and follow the National Standards for Bereavement Care (HSE 2022), which require the CMS in bereavement and ensure staff access to training and support. Differences in hospital size may influence midwives' exposure to perinatal loss and access to resources, enabling the study to capture a broad range of bereavement care experiences in Ireland's public maternity services.

Participants were recruited between July and November 2024 using purposive sampling (Etikan 2016) and snowball sampling (Sadler et al. 2010). Following approval from the director of midwifery at each site, recruitment flyers were displayed in staff areas (e.g., break rooms and notice boards) by the designated gatekeeper. The flyers outlined the study objectives, eligibility criteria (Table 1), the contact details of the first author, and included a QR code linking to an online registration form. Interested midwives scanned the QR code to access the eligibility screening questions and to provide their preferred contact details. Direct experience of providing perinatal bereavement care was not required for inclusion, as the study sought to capture a wide range of midwives' perceptions and experiences of providing PSIs within maternity care. Eligible participants were contacted by the first author and sent the participant information sheet and consent form.

**TABLE 1:** Eligibility criteria of participants.

Inclusion criteria	Exclusion criteria
Being a registered midwife—have a minimum of one‐year clinical experience in Maternity Services in Ireland. Aged 18 years old or above. Sufficient ability to speak, read and write English. Capacity to provide informed consent.	Student midwives. Have not provided informed consent.

Written informed consent was obtained via email for online interviews and in person for face‐to‐face interviews. After providing signed consent forms, participants completed the online questionnaire (QuestionPro: https://www.questionpro.com/) to collect demographic information before the interview. The questionnaire also included the 15‐item self‐report Evidence‐Based Practice Attitude Scale (EBPAS), which has good reliability and internal consistency (Cronbach's alpha 0.76) (Aarons 2004). The EBPAS assesses the attitudes towards the adoption of evidence‐based practices (PSIs) across four domains (Aarons et al. 2010): (1) openness to implementing new interventions (Openness); (2) the intuitive appeal of the new intervention (Appeal); (3) willingness to use required interventions (Requirements) and (4) perceived conflict between clinical experience and research evidence (Divergence). Items are rated on a 5‐point Likert scale (0 = Not at all to 4 = To a very great extent), with subscale scores contributing to a total score. For this study, the mean total measure score was calculated to describe the participants' attitudes towards adopting new PSIs to support bereaved parents, as part of the sociodemographic background information.

### 4.3 Ethical Considerations

Ethical approval was obtained from the Research Ethics Committee at the University of Galway (Reference number: 2024.03.022) and from three Research Ethics Committees of participating hospital sites in Ireland (Reference numbers: Dublin REC‐2024‐010, Galway C.A.3163, and Limerick 014/2024). All participants were provided with written and verbal information about the study and the voluntary nature of participation. Their rights were explained in terms of confidentiality and anonymity of data collected and all participants provided signed informed consent before each interview. All data were stored in a password‐protected cloud database for data protection, accessible only to the research team.

### 4.4 Data Collection

The development of the semi‐structured interview topic guide was informed by our integrative review (Xie, Hunter, et al. 2022; Xie, Tang, et al. 2022), suggestions from the steering group and further refined following three pilot interviews with two midwives. Revisions removed questions that were repetitive, improved the logical flow of questions, and enhanced the sensitivity of the language used. The final interview guides (Data S3) were reviewed and approved by the steering group.

Individual semi‐structured interviews were conducted via Microsoft Teams, Zoom or in person, depending on participant availability and preferences. All interviews were conducted on a one‐to‐one basis with no other individuals present. No prior relationship was established between the researcher and participants before the study commenced. At the beginning of each interview, the researcher introduced herself and explained the study objectives. All interviews were audio‐ or video‐recorded with participants' consent and lasted approximately 60 min on average. Field notes on interview interactions were also made by the first author during and after each interview, which also added to the analytical process.

### 4.5 Data Analysis

Data analysis was conducted concurrently with data collection. Interviews were transcribed verbatim by an external agency, pseudonymised (e.g., M01) and anonymised before being imported into NVivo (Version 20) by the first author (JX) for coding and organisation. Descriptive statistics were generated to summarise the sociodemographic characteristics of participants using IBM SPSS Statistics (Version 23.0). No missing data were identified.

Data analysis was led by JX and AH, with regular input from the other two authors (AMG and LB) whose expertise spans qualitative research, perinatal mental health, PSIs, and midwifery research. Braun and Clarke's (2021) reflexive thematic analysis (RTA) was employed, guided by their six‐step framework (Braun and Clarke 2006), using a data‐driven approach to identify and interpret patterns within the data. This six‐step framework was applied: (1) the first author read each interview transcript repeatedly to gain an overall impression, taking notes and analytic memos during the process; (2) Coding was conducted in NVivo using an inductive approach, capturing semantic and latent meanings line by line across the dataset; (3) related codes were grouped into themes through an iterative process in Excel, with visual mapping to ensure they reflected the breadth and depth of the data; (4) initial themes were reviewed and discussed within the research team to ensure internal coherence and clear distinctions; (5) themes were defined and named during research team meetings; and (6) themes were written up with illustrative verbatim quotations selected for their representativeness and ability to convey the essence of each theme. RTA (Braun and Clarke 2021) treats researcher subjectivity as an analytic tool, prompting the first author (JX) to reflect on her values, experiences, and assumptions about knowledge generation and research practice.

### 4.6 Rigour and Reflexivity

To ensure methodological and analytical rigour of this study, the four criteria of trustworthiness in qualitative research defined by Lincoln and Guba (1985) were employed: credibility, transferability, dependability, and confirmability. The strategy and action taken by all authors are listed in Table 2, including how authors engaged in in‐depth discussions, critiqued each other's interpretations of the dataset and how all ambiguities related to the themes/subthemes were thoroughly discussed and resolved collaboratively between all authors.

**TABLE 2:** Trustworthiness of the qualitative data as recommended by Lincoln and Guba (1985).

Quality criteria	Action taken by the researchers
Credibility	Regular research team meetings were conducted throughout the data collecting and analytic process to facilitate problem‐solving, coding alignment, and theme refinement. Feedback was sought from steering group members with relevant lived experiences about identified themes and subthemes to enhance interpretive rigour.
Transferability	Detailed accounts of the research context, participants, and findings were offered so that readers can assess the applicability of the results to other settings.
Dependability	A detailed record was maintained throughout the data generation process.
Confirmability	All researchers were involved in analysis and an audit trail of data analysis were kept in research team.

In addition, reflexivity was actively integrated throughout the research process to enhance rigour. The first author, who conducted the interviews, is a female PhD student in Nursing with a background in mental health and maternity care research and formal training in qualitative research, interview techniques, and RTA. Although not a midwife and without personal experience of perinatal loss, the author's clinical and academic background informed a sensitivity to the emotional dimensions of bereavement care. The first author maintained a reflective journal to continually examine her experiences, assumptions, and potential influences on data interpretation. Adopting reflexivity during data analysis (Peddle 2022) helped ensure the analysis remained grounded in participants' accounts, with codes and themes generated free from pre‐conceived ideas.

## 5 Results

### 5.1 Characteristics of Participants

A total of 25 midwives were approached. One midwife who did not meet the minimum requirement of 1 year of clinical experience was deemed ineligible. Two midwives expressed initial interest but did not provide consent after receiving the study information and were therefore excluded. The final sample comprised 22 female midwives: six from Site 1, 10 from Site 2, and six from Site 3. Participants' professional experience ranged from 2 to 37 years, and covered practice areas including antenatal care, labour and birth, bereavement support, and leadership roles. All participants had experience in providing bereavement care, and the vast majority (18/22) had received relevant training (see Data S4 for individual participant characteristics). Confidence in delivering PSIs ranged from completely to somewhat confident. The mean EBPAS (45.36) indicated a generally positive attitude towards evidence‐based practice. The subscales suggested openness to new interventions, intuitive appeal of evidence‐based practice, moderate willingness to adopt it when required, and perceived consistency with clinical experience.

Overall, the group represented an experienced cohort of midwives with varied clinical roles across three maternity sites, encompassing both senior and early‐career practitioners who demonstrated openness to evidence‐based PSIs. A summary of sociodemographic characteristics is presented in Table 3.

**TABLE 3:** Characteristics of participants.

Characteristics of participants (total *n* = 22)	Mean	Standard deviation (range)
Age	40.68	2.37 (24–58)
Years of working experience	14.95	2.32 (2–37)
Evidence‐Based Practice Attitude Scale	45.36	7.40
Openness	11.77	2.89
Appeal	13.27	2.53
Requirements	8.36	3.17
Divergence	11.95	2.55

	Frequency *n* (%)
Gender	
Female	22 (100.00%)
Nationality	
Irish	19 (95.00%)
Prefer not to answer	1 (5.00%)
Highest education	
Higher diploma	1 (4.55%)
Bachelor's degree	11 (50.00%)
Master's degree	10 (45.45%)
Current practice area	
Antenatal care	7 (31.82%)
Labour and birth	3 (13.64%)
Gynaecological unit	1 (4.45%)
Manager/leader	2 (9.09%)
Bereavement support midwife	3 (13.64%)
Other	6 (27.27%)
Bereavement care experience	
Yes	22 (100.00%)
Bereavement training	
Yes	18 (81.82%)
No	3 (13.64%)
Prefer not to answer	1 (4.55%)
Confidence in providing PSI	
Completely confident	8 (36.36%)
Fairly confident	10 (45.45%)
Somewhat confident	4 (18.18%)

### 5.2 Interview Findings

**FIGURE 1:**
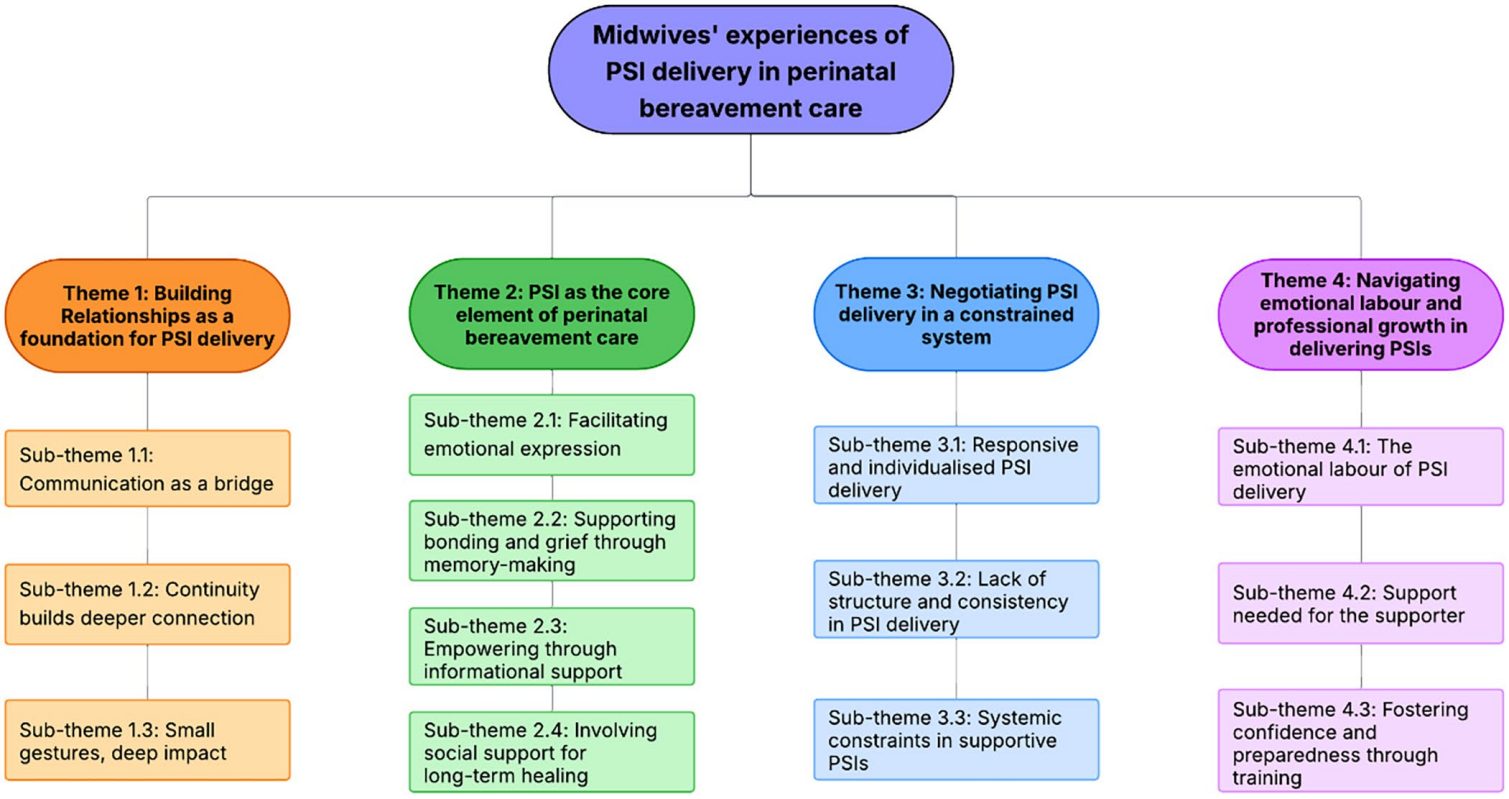
Thematic map.

Four themes and their supporting subthemes (Figure 1) were generated to reflect midwives' perceptions and experiences of providing PSIs to parents following perinatal bereavement in the Irish maternity care setting: (1) Building relationships as a foundation for PSI delivery; (2) PSI as the core element of perinatal bereavement care; (3) Negotiating PSI delivery in a constrained system; (4) Navigating emotional labour and professional growth in delivering PSIs. These themes and subthemes are discussed in detail below with illustrative supporting quotes (Data S5), showing how these experiences contribute to understanding and optimising PSI delivery in perinatal bereavement care.

#### 5.2.1 Theme 1: Building Relationships as a Foundation for PSI Delivery

The data indicates that the starting point for meaningful PSI lies in the relationship between the midwife and the bereaved parents. This theme illustrates how midwives' perceptions of how trust and compassionate connection create the conditions in which PSI can be delivered. The theme shows that relationships are built upon a series of caring and consistent interactions, such as effective communication, continuity of care, and compassionate gestures, all of which help parents feel well cared for and supported during their grief.

##### 5.2.1.1 Communication as a Bridge

In the emotionally charged context of perinatal bereavement care, midwives described communication as a vital means of fostering emotional connection and trust. They emphasised that inclusive communication with both parents was crucial for validating grief and supporting the emotional needs of the family as a whole. Thoughtful language, offering condolences, acknowledging the loss, open‐ended questions and sensitively explaining care plans, were seen as central to helping parents feel supported and understood. Midwives noted that parents often remembered not the clinical procedures, but the compassionate communication they received:

When the parents are asked about their care…it's not primarily the physical care they'll come back and talk about, it will be the communication, it will be the compassion…and making that authentic connection, it's so important. (M18)

However, communication was not always straightforward. Midwives described emotional and practical barriers, including fear of saying the wrong thing, parental anger, and language differences, which could make empathetic communication more difficult to sustain:

I find that when they're angry…they'll question everything…it's really difficult to communicate with them and try and make them at ease and support them. (M10)

##### 5.2.1.2 Continuity of Care Builds Deeper Connection

Continuity of care enabled midwives to build trust, emotional safety, and provide personalised support, particularly vital in the vulnerable context of perinatal loss. Ongoing engagement fostered rapport and emotionally attuned care, not merely through task consistency, but through relational depth that helped buffer the isolation of grief.

The patients that I have met at the time of their diagnosis of their loss, I find that I have more of a connection from the beginning…they tell me that it helps them to speak to me after. (M18)

Yet, the ideal of continuity was often disrupted by structural challenges such as rotating shifts or weekend staffing gaps. Midwives emphasised that clear documentation and handovers were essential for maintaining a sense of continuity across providers:

We can write everything down into the chart, so we are not asking the women the same questions forty times…it's continuity for the midwife (M20)

Midwives frequently described breaks in continuity, particularly during weekends or holidays, as significant barriers to care. The absence of a CMS in bereavement during these times was seen to disrupt emotional support and heighten strain for both families and staff. To address this, some participants advocated for 7 days of bereavement support to ensure families receive specialised care regardless of when the loss occurs, while also alleviating pressure on midwives who may feel unprepared for this role. However, others cautioned that over‐reliance on a specialist could deskill generalist midwives and weaken the wider team's capacity for compassionate care:

If you have a clinical midwife specialist answering a bleep 24/7 to come to a patient, my worry about that is would the midwives lose their skills? (M18)

##### 5.2.1.3 Small Gestures, Deep Impact

Midwives emphasised that small, consistent gestures can have a powerful impact on the emotional experience of grieving parents. Simple acts, such as meeting parents at the door, introducing themselves clearly, and providing a cup of tea, were seen as crucial for establishing trust and emotional safety. Meanwhile, midwives highlighted approachability and emotional presence as critical to maintaining rapport:

Being the midwife caring for these patients, being approachable and just letting them know that you're there to support them through it. That they feel safe when you're there. (M07)

Actions, such as holding a hand, giving a hug, or offering tissues, blanket or food, were also described as powerful forms of build relationship. Through these small gestures, midwives cultivated an atmosphere of emotional safety and trust through their presence, rather than through clinical or procedural tasks:

My natural reaction would be to provide some form of comfort, you know hold their hand, give them some tissues, touch their back, if they feel like they need a hug or whatever. (M22)

Beyond direct interactions, midwives described using known symbols as cues within the care environment to promote collective empathy. For example, an end‐of‐life sign served as a subtle yet powerful reminder for staff to maintain a respectful, quiet atmosphere:

We do put up the end‐of‐life sign…so everyone then like lowers their voices, they speak quieter, we lower the fetal monitors down. It just gets very respectful if that sign is up. (M07)

#### 5.2.2 Theme 2: PSI as the Core Element of Perinatal Bereavement Care

Once a relationship is established, midwives described how PSIs become the central thread of bereavement care. This theme reflects midwives' experiences of implementing specific PSIs as a central component of bereavement care, highlighting practices that support parents' emotional expression, memory‐making, empowerment, and engagement with wider social support networks. These practices formed a scaffold of care that aimed not only to comfort parents in hospital, but to empower them in their long‐term grieving process.

##### 5.2.2.1 Facilitating Emotional Expression

Midwives saw their role as providing emotional as well as physical support by creating safe spaces where grief could be openly expressed. This sub‐theme centres on the idea that healing begins when parents are allowed to grieve without fear of judgement. Key to this was normalising grief, openly acknowledging the loss, and validating parents' emotions, whatever they may be, without avoidance or minimisation.

Most people are just going through grief and loss. So it's important that we realise that and we normalise the feelings of grief and loss. (M19)

Midwives facilitated emotional expression through compassionate presence, being physically and emotionally available, listening without judgement, and allowing space for grief without trying to fix it. Presence was often about simply “being” rather than “doing”, especially in silence. Several midwives described the therapeutic value of sitting in silence with parents, resisting the urge to speak or provide quick reassurance:

That's the first stages of grief, the silence. And you have to be comfortable rather than filling the silence with rubbish. (M02)

Midwives also described using embodied methods to help parents process and express grief. These included journaling, drawing, and letter writing that helped externalise emotions and create meaning:

Writing a letter to the baby, to go into the coffin with the baby, that's something that I've seen has been helpful a few times. (M12)

In addition, midwives used mindfulness, relaxation, and sensory‐based strategies to help parents regulate distress and access emotional calm:

Meditation, visualisation of where a happy place could be for them, and box breathing…I find it works amazing (M15)

##### 5.2.2.2 Supporting Bonding and Grief Through Memory‐Making

Midwives described memory‐making and honouring the baby as meaningful components of bereavement care. These practices not only help families navigate their loss, but also give midwives a sense of purpose and fulfilment in their caregiving.

Recognising the baby as a person, by using their name, saying “congratulations,” noting their features, or encouraging parents to spend time with them, was a powerful way to affirm the baby's life and the parents' identity. Acts such as holding, bathing, or dressing the baby were seen as vital for fostering emotional connection and supporting grief.

It's very important I think to recognise features of the baby…that's their story then for their baby…that's how you make memories and that's how they get to know their baby. (M15)

Memory‐making activities, such as hand and footprints, locks of hair, memory boxes, and birthstones, were described as small but profoundly meaningful:

The memory making is probably one of the biggest things…it makes a connection, and it's also inclusive of the family. (M21)

As parents navigated their grief, midwives acted as “gatekeepers” of memory‐making, offering gentle encouragement and reassurance while respecting parental autonomy. They also emphasised the importance of allowing space for change, recognising that decisions made in the moment might shift with time and support:

I did also meet women who didn't want photographs, and I suppose we have to accept that too…letting them know that… just because you said no at ten o'clock doesn't mean you can't change your mind at two. (M02)

##### 5.2.2.3 Empowering Through Informational Support

Midwives highlighted that clear, consistent, and sensitively delivered information can restore parents' sense of agency and reduce anxiety during a disorienting time. As many parents are in emotional shock, even well‐intentioned communication can feel overwhelming. Midwives therefore advocated pacing verbal and written information, presenting it in manageable amounts, and allowing time for emotional processing. Information was seen not just as a way to convey care options, but as a tool for empowerment, helping parents regain a sense of control and choice when much feels lost:

It's about not overloading them with everything at once because they can't take that in…You're not rushing them making decisions, you're giving them time to talk about it, to think about it, for the idea to develop with them. (M03)

Recognising that distress can limit parents' ability to absorb details, midwives stressed the need to reinforce key messages over time, adopt a staged approach, and provide follow‐up conversations:

I usually just find out from the parents where they are and find the helpful things that they already have or help them to notice the important things that they already have. (M18)

At a systemic level, midwives were aware that inconsistent or conflicting information could undermine trust and intensify parents' distress. They described the need for alignment and communication across the multidisciplinary team:

I suppose in a busy hospital things go get missed…it's about sharing the communication…it isn't just us going around, it's the porters, the catering, the household, you know the electricians, the midwives, nurses, doctors, consultants—it's everyone's responsibility. (M21)

These insights demonstrate that midwives viewed informational support as an extension of compassion, helping parents feel less lost, better informed, and more in control of their own journey through grief.

##### 5.2.2.4 Involving Social Support for Long‐Term Healing

Midwives described their role in encouraging bereaved parents involves activating a wider circle of emotional, social, and cultural resources to reduce isolation and strengthen long‐term coping.

Acknowledging the eventual limits of their own involvement, they emphasised the importance of helping parents draw on support networks such as partners, siblings, extended family, and close friends:

You bend the rules and make sure she has other support with her. Like her partner or her sister or mother…you try and help to bridge that gap because you can't be everywhere—that's the reality of it. (M06)

Supporting the wider family also meant helping parents communicate the loss to siblings, particularly young children. Midwives suggested using practical tools—such as counselling, books, and keepsakes, to guide these difficult conversations and foster collective remembrance:

I think counsellors that are appropriately trained and you know giving resources like maybe books and talk to them about how to explain to the sibling what has happened. (M03)

Spiritual care emerged as another valuable component of social support. Midwives often facilitated access to chaplains or religious figures, while remaining sensitive to the cultural and spiritual diversity of bereaved families:

The chaplain support works very well…[but] it's not for everybody, some people aren't religious and obviously we are a multicultural society now. (M12)

Looking beyond the hospital, midwives also signposted parents to peer support groups and bereavement charities. These services helped bridge the emotional gap after discharge and offered parents opportunities for continued support:

Because you are not alone, while everyone's situation is unique you are not the only one who has suffered and there might be some support in being like it's not just me. (M04)

By engaging wider support networks, midwives offered more than short‐term care, they helped parents build a foundation for long‐term healing and supported families in grieving in ways that felt both culturally and emotionally meaningful.

#### 5.2.3 Theme 3: Negotiating PSI Delivery in a Constrained System

Despite the central role of PSIs, midwives often faced systemic and structural barriers to their delivery. This theme describes the challenges midwives encountered while they continuously adapt and balance their emotional presence in response to parents' needs. Time pressures, staff shortages, limited resources, and the absence of formal frameworks frequently constrained their efforts. While midwives valued individualised care, they also emphasised the need for structured guidance to ensure consistent and equitable PSI delivery across settings.

##### 5.2.3.1 *Responsive* and Individualised PSI Delivery

This sub‐theme illustrates how midwives framed PSI delivery as responsive and guided by parents' emotional cues, preferences, and cultural contexts rather than fixed protocols. Emotional responsiveness involved not just observation, but actively deferring to parents' signals. In this co‐constructed process, support is guided by an ongoing, often unspoken dialogue in which midwives listen, adapt, and affirm parents' agency:

I will go by their cues…it's the couples lead you basically. It's not a one shoe fits all, couples will tell you most of the time what they need…if they don't tell you, if you ask, they will let you know. (M07)

This commitment to individualised care was also evident in how midwives offered PSIs. Rather than pressuring parents, they focused on gently offering and encouraging options they believed could support long‐term healing

We can't force them. All we can do is offer them and encourage them if we feel they will bring long‐term benefit and healing. (M02)

Midwives described offering support as an ongoing process rather than a one‐time event. Parents were given space to revisit decisions throughout the day, free from pressure or judgement. Midwives also highlighted the importance of adapting their approach to reflect diverse cultural practices and emotional norms, reflecting a culturally sensitive understanding of bereavement care:

The big thing is respecting everyone else has different wishes and everyone's culture and custom and norms. (M11)

##### 5.2.3.2 Lack of Structure and Consistency in PSI Delivery

This sub‐theme explains midwives' response to the lack of clear guidelines, relying instead on experience, personal judgement, and instinct. Some midwives stated they worried that such variability could lead to unequal experiences for bereaved parents:

In terms of guiding how our interaction is, there isn't really a major guide as such. It's kind of just your own personal, what you feel works. (M08)

Concerns were particularly strong regarding newly qualified or less experienced staff, who often had to navigate bereavement care without formal training or structured support. They stressed that a structured approach would benefit both staff and parents, not by imposing rigid protocols, but by offering supportive frameworks that promote consistency while allowing for relational flexibility. Midwives highlighted the need for flexible, practice‐informed tools to help them meet parents' needs, reduce emotional risk, and ensure equitable care.

It would be great if there was a framework designed that…we could support women with that…that would support the midwife as well. (M03)

##### 5.2.3.3 Systemic Constraints in Supportive PSIs

Midwives faced multiple constraints that hindered PSI delivery. Heavy workloads, staff shortages, and time pressures limited their availability and often left them emotionally distressed when they were unable to provide the level of care they felt was needed.

There's a huge amount of paperwork…you may not have as much time as you'd like to deal with the emotional side of it. (M03)

It really affects us because that's when we'll go home upset, saying there was a woman who was bereaved, and I feel I didn't spend the time with her. (M15)

The physical environment also played a critical role. Having access to a dedicated space, a private room with an ensuite, was described as ideal for both parents and midwives, as it made midwives feel that parents were receiving a better service. However, the proximity of bereaved parents to the joyful sounds of birth and celebration added to their distress:

Next door has a baby crying, and people cheering…her heart is broken in the bed. (M09)

To ease this emotional strain, midwives used creative strategies—such as playing background music—to soften the sensory environment and create a calmer space. However, these efforts were often constrained by resource gaps. Limited access to memory‐making tools left midwives reliant on charities and feeling helpless when essential items were unavailable:

We need more cuddle cots from Féileacáin…we need more of everything. (M18)

Within these systemic limitations, midwives also recognised the boundaries of their own professional role: “I'm not trained to be a therapist, I'm trained to be a midwife”. This lack of specialised training was compounded by limited access to appropriate psychological support. As one midwife explained, mental health services often failed to recognise bereavement as a legitimate referral reason, leaving families without appropriate support:

It's very hard to find what is mental health and what is bereavement…Mental health department won't see those families because they don't see bereavement as a mental health (M21)

#### 5.2.4 Theme 4: Navigating Emotional Labour and Professional Growth in Delivering PSIs

This theme turns inward to capture midwives' experiences of the emotional demands of PSI delivery and the resources they require to sustain their practice. Their ability to deliver high‐quality PSIs was shaped not just by external resources, but by their emotional readiness. Peer support, reflective opportunities, and tailored training were seen as essential to maintaining compassion and resilience.

##### 5.2.4.1 The Emotional Labour of PSI Delivery

Midwives described providing psychosocial support as a distinct form of emotional labour: demanding, often invisible work that requires emotional presence, self‐regulation, and the ability to hold space for parents' grief. They reflected on the personal cost of being emotionally available, noting that while support was deeply valued by families, it could also feel depleting:

They really value if you're there emotionally to support them. That can be draining, because sometimes you feel oh gosh, I've given so much of myself to that woman. (M03)

Rapid switching between emotional states, from grief to joyful maternity care, added to the emotional burden, which was rarely acknowledged in formal systems:

Your tone, your pace changed, everything changed. That is really emotionally draining…it's a real roller coaster of emotions for a midwife. (M05)

Some experienced midwives were repeatedly assigned to bereaved families, while this reflected trust in their skill, it also contributed to emotional fatigue:

I'm doing this all the time, and I can't do anymore. (M03)

Even though participants described an effort to actively set boundaries to avoid burnout, these emotional boundaries were not static; they were constantly being renegotiated, especially when midwives' personal histories of loss surfaced in their professional encounters. Personal histories of loss could deepen empathy but also triggered emotional strain:

It could be triggering to look after bereaved parents…it can impact on your own mental health. (M12)

##### 5.2.4.2 Support Needed for the Supporter

While PSI was described as profoundly meaningful, midwives emphasised that sustaining this work requires consistent support, which is often lacking. Midwives emphasised that emotional support should be a shared professional and institutional responsibility. Without proper support, midwives were left carrying emotional burdens alone:

I find I kind of feel drained…emotionally and psychologically…and I kind of feel, well where do I go for support? And there's no support for the midwives. (M03)

Existing options like counselling were seen as inaccessible or culturally mismatched. Midwives reflected on the structural absence of institutional support tailored to the unique emotional toll of bereavement care:

There is a support number here…but nobody ever rings it. How can you ring and say, ‘I was minding a baby that has died, and I'm upset?’ Like what do they even say? (M15)

Participants also valued supportive managers who adjusted workloads accordingly. Meanwhile, colleagues' support was described as essential, offering emotional safety, shared understanding, and backup when needed.

You don't even have to say anything, you just start crying…we are very supportive of each other. (M12)

In addition, the CMS in bereavement is identified as an important support resource, providing emotional and practical guidance and often being the “go‐to” person for midwives:

When she would come to the ward, I remember distinctly her first question to me was ‘how are you doing?’ She was a massive support to the midwives…she was our go‐to person. (M02)

Participants called for routine and non‐judgemental reflection, not just debriefing after adverse events, but ongoing emotional support and experiences sharing through peer support group:

A peer group for midwives…we could check in on each other in a safe environment to discuss how that went and how we felt and what worked well and what we could improve. (M03)

This sub‐theme reveals that midwives didn't seek to avoid emotional labour, they wanted to be supported within it, so they could remain emotionally available to bereaved parents:

If the carer isn't being looked after, they may not be giving the best care. (M02)

##### 5.2.4.3 Fostering Confidence and Competence Through Training

This sub‐theme captures midwives' needs for training that prepares them for the emotional and relational complexities of bereavement care. Many entered practice with little or no preparation, leaving them uncertain and afraid of saying or doing the wrong thing:

The challenge…is we have a fear that we'll say something wrong or do something wrong. (M03)

I had to learn by maybe making someone else uncomfortable…but having some training would have made it easier. (M04)

While procedural training was common, participants called for practical training like handling a deceased infant, and emotionally focused training, such as how to sit in silence, handle a deceased infant, and support grief:

Some people aren't very good at going in and just letting someone cry and talk…there could be more emotional support kind of study day. (M13)

Training was also viewed as a way to ensure consistency in care. Without it, bereavement support risked falling to a small group of more experienced or emotionally attuned midwives:

It's important that all midwives are skilled in the interventions…and it's not just left to one or two or three midwives on the floor. (M03)

The aim of training is to help midwives build a flexible “toolbox” of emotional and practical strategies to support bereaved parents. This toolbox gave them the confidence to respond with parents' grief and provide compassionate care to help parents through the grieving process.

The more resources we have in our toolbox to be able to respond to parents' needs…the better we will be able to do our job, and minimise the traumatic effect for the parents. (M03)

## 6 Discussion

This study explored midwives' perceptions and experiences of delivering PSIs to parents following perinatal bereavement in the Irish maternity care setting. The four themes identified show the complexity and emotional depth of PSI delivery in perinatal bereavement care. As a means of applying the identified themes to clinical reality, the domains of the Socio‐Ecological Model (SEM) (McLeroy et al. 1988) guided the structure for organising and contextualising the findings, highlighting how individual, interpersonal, institutional, community, and policy‐level factors influence PSI delivery. The section below will interpret the findings through this model, allowing a targeted understanding of midwives' experiences and providing a strong foundation for informing the future design of a structured midwife‐led PSI.

### 6.1 Individual Level: Emotional Preparedness and Professional Commitment

At the individual level, participant midwives demonstrated deep personal commitment to supporting bereaved parents but reported variable confidence in their ability to deliver PSIs and limited formal preparation, as illustrated in Characteristics of participants, theme 3 and theme 4. These findings align with previous studies showing that midwives frequently rely on intuition, peer observation, or experiential learning when providing bereavement care (Garcia‐Catena et al. 2023; Ravaldi et al. 2018). Theme 4 shows that less experienced midwives described anxiety about saying the wrong thing, reflecting the internal conflict between compassionate intention and emotional uncertainty. This supports Power et al. (2022), who found that emotional unpreparedness is a significant source of distress and hesitation among midwives providing bereavement care.

Our data advances this understanding by showing a clear desire for emotionally focused training that goes beyond clinical tasks to encompass presence, silence, and grief literacy, areas often underemphasised in traditional bereavement education (Aggarwal and Moatti 2022). Echoing Qian et al. (2022), midwives in this study called for training to be rooted in real‐world emotional complexity, preparing them to engage with parents' grief without becoming emotionally overwhelmed. SEM highlights that while individual skill building is essential, it must be supported by changes at higher levels, such as institutional and policy levels, to ensure sustainability.

### 6.2 Interpersonal Level: Building Relational Foundations for PSI Delivery

Interpersonal relationships formed the foundation for effective PSI delivery. The midwives' role in facilitating emotional expression, memory‐making, informational empowerment, and social integration was central at this level, as illustrated by themes 1 and 2. Consistent with Dolan et al. (2022) and Ravaldi et al. (2018), midwives in our study viewed compassionate, trust‐based relationships as foundational to meaningful support. Theme 1 illustrates how continuity of care, compassionate communication and small acts of kindness create the emotional conditions in which PSIs can be meaningfully enacted. These relational elements align with previous research indicating that meaningful connections and empathetic communication significantly enhance bereaved parents' psychological adjustment (Cote‐Arsenault and Denney‐Koelsch 2016). The national standards (HSE 2022), also recommend relational continuity and empathetic communication as key components of bereavement care. Continuity of care was conceptualised as a structural consideration but as a relational imperative to establish trust, consistent with findings from Fenwick et al. (2007), highlighting the stabilising effects of continuous caregiver relationships on grieving families.

However, midwives also encountered relational challenges, including language barriers, parental anger, and disrupted continuity due to structural realities. Maintaining relational continuity was highlighted as critical yet difficult, often impeded by staffing patterns and resource limitations. From an SEM perspective, these findings underline the need for enhancing relational, cultural competence and advanced communication training to help midwives navigate emotionally complex interactions while maintaining rapport and trust (Gold 2007; McNamara et al. 2022).

### 6.3 Institutional Level: Balancing Compassionate Intent With Systemic Constraint

At the organisational level, midwives described an ongoing tension between their commitment to delivering PSI and the structural limitations of their working environments. Theme 3 highlights how staffing shortages, inadequate physical space and resources, lack of standardised guidance, and competing clinical demands inhibit consistent support and negatively impact midwives' emotional wellbeing. These findings echo those of Due et al. (2018) and Nash et al. (2018), who also found that the capacity to deliver bereavement support was undermined by clinical constraints.

Theme. 3 also identifies the importance of supportive frameworks for midwives. This finding is echoed by Ravaldi et al. (2022), who suggest that strong application of clinical guidelines around staff support can enhance midwives' sense of personal accomplishment and reduce burnout. However, midwives in our study expressed a clear preference for adaptability over rigid protocols, emphasising the need for flexible and evidence‐informed guidelines that support PSI delivery. Such guidelines can help midwives not only to provide consistently high‐quality care with the same standard but also allow them to respect the individuality and diversity of parents' grief.

A particularly notable institutional finding was the concern between specialist and generalist midwives. The CMS in bereavement were valued for their expertise and support for both bereaved parents and midwives, and this role was highly recommended to be integrated into health institutions globally (Samoah et al. 2023). However, our finding highlights concerns that over‐reliance on bereavement specialist midwives may inadvertently deskill generalist midwives. If the delivery of PSIs is overly concentrated in specialist roles, broader midwifery workforce competencies might diminish, and more importantly, may disrupt continuity of care across the maternity service. Institutional strategies should thus balance specialist expertise with widespread capacity‐building, fostering generalist midwives' PSI delivery competencies.

To address the emotional labour inherent in bereavement care, institutions must provide training and targeted psychological support for midwives caring for bereaved families. Such structured support is critical not only for mitigating burnout but also for sustaining high‐quality PSI delivery. This aligns with Homer et al. (2016), who argue that healthcare providers need training to ensure that they are equipped to provide appropriate care and should have access to debriefing and professional support. When these supports are lacking due to resource constraints, the emotional toll is amplified, for both the bereaved parents and the clinicians supporting them. SEM analysis underscores that institutional‐level interventions, such as formal frameworks, structured training, and protected time, are critical to enabling individual and interpersonal‐level skills to be enacted in practice.

### 6.4 Community Level: Mobilising Social and Cultural Networks

Midwives recognised the critical role of community‐level influences on bereavement experiences. Theme 2 indicates that providing meaningful PSI was seen to require sensitivity to cultural and religious diversity, as well as the inclusion of parents' broader social networks, such as family members, peer support groups, and voluntary organisations. These wider community support systems were viewed as essential for sustaining PSI delivery beyond the immediate clinical encounter. This perspective aligns with Kingdon et al. (2019), who identified enduring gaps in culturally responsive care and called for more integrated and equity‐focused strategies, suggesting that greater community engagement in bereavement care may help address these gaps. In practice, midwives in our study often acted as facilitators, signposting parents to peer support groups, supporting sibling inclusion, and offering culturally respectful referrals to chaplains or community leaders. However, the ability of the midwives in this study to consistently activate these resources was constrained by limited time and unclear referral pathways. To support midwives in this role, structured referral systems and communication protocols may promote community engagement and enable culturally responsive and holistic care (Connell 2019). From an SEM perspective, community‐level action is a crucial but underdeveloped area of PSI delivery. Strengthening formal partnerships with community and voluntary organisations could improve continuity of support and address cultural needs, complementing interventions at the institutional and policy levels.

### 6.5 Policy Level: Bridging Standards and Practice Through Structured PSI Frameworks

Although national standards in Ireland (HSE 2022) recognise the importance of psychosocial support in perinatal bereavement, theme 3 highlights the absence of specific operational frameworks to guide its implementation in daily practice. Similarly, Martinez‐Serrano et al. (2018) found that midwives frequently navigated care without formal guidance, relying instead on personal judgement and informal team norms. This lack of structured guidance contributes to variability in care quality, making the delivery of PSIs inconsistent and largely dependent on individual midwives' initiative. To address this gap, policy‐level interventions must move beyond general recognition of need to the establishment of clear, actionable frameworks. These should include evidence‐informed guidelines, defined care pathways, accountability structures, dedicated funding streams, and mandated training standards. Embedding PSIs explicitly within national maternity care policy would promote consistent, equitable, and sustainable provision of PSI across settings, helping to move this aspect of care from discretionary practice to core service delivery.

### 6.6 Strengths and Limitations

This study offers valuable insights into midwives' experiences of delivering PSIs in perinatal bereavement care across varied maternity settings in Ireland. The descriptive qualitative design, reflexive thematic analysis, and inclusion of midwives from three service contexts, alongside Patient and Public Involvement, enhanced the depth, credibility, and relevance of the findings. Applying the SEM throughout the Discussion provided a multi‐level understanding of influences on PSI delivery and clear targets for practice and policy. Researcher reflexivity was actively maintained, with positionality recognised as an analytic strength.

Limitations relate to the study's scope. As in most qualitative research, findings are contextually situated and not intended for statistical generalisation (Maxwell 2021). The sample may overrepresent midwives with a strong interest in bereavement care, potentially underrepresenting less confident perspectives. As this phase focused solely on midwives' views, future work should incorporate bereaved parents and other stakeholders to support inclusive intervention co‐design.

### 6.7 Recommendations for Further Research

Future research should examine bereaved parents' experiences of receiving PSIs during perinatal loss to identify what they find meaningful, distressing, or lacking. Integrating these perspectives with midwives' views will enable co‐designed interventions that are emotionally relevant, responsive to parental needs, and feasible within routine maternity care.

### 6.8 Implications for Policy and Practice

Strengthening midwife‐led PSIs could enhance the quality, consistency, and equity of bereavement care. National standards should explicitly include PSIs as core components, supported by clear guidance, sustainable funding, and workforce development. For clinical midwives, this means access to training, confidence‐building opportunities, and peer support. Organisationally, maternity services should provide protected time, suitable spaces, and staff support structures to sustain compassionate, responsive care.

## 7 Conclusion

This study explored midwives' perceptions and experiences of providing PSIs to support parents through grief following perinatal bereavement in Ireland. Interpreted through the SEM, the findings show that PSI delivery is influenced by factors at the individual, interpersonal, institutional, community, and policy levels. Applying SEM provided a multi‐level understanding of barriers and enablers, identifying leverage points for developing contextually grounded, midwife‐led interventions. Future efforts should focus on embedding PSIs within national maternity policy, developing institutional frameworks for training and support, fostering community partnerships, and strengthening relational and cultural competence.

## Author Contributions

**Jiaying Xie:** conceptualisation, formal analysis, investigation, data curation, writing – original draft preparation, writing – reviewing and editing, visualisation, project administration. Dr. **Annmarie Grealish:** conceptualisation, formal analysis, investigation, validation, supervision, writing – reviewing and editing. Dr. **Linda Biesty:** conceptualisation, formal analysis, investigation, validation, supervision, writing – reviewing and editing. Dr. **Andrew Hunter:** conceptualisation, formal analysis, investigation, data curation, writing – original draft preparation, writing – reviewing and editing, visualisation, project administration, funding acquisition.

## Ethics Statement

This study received ethical approval from the Research Ethics Committee at the University of Galway (Reference Number: 2024.03.022). Ethical approval was also obtained from the three Research Ethics Committees of participating hospital sites in Ireland (Reference numbers: Dublin REC‐2024‐010, Galway C.A.3163, and Limerick 014/2024).

## Consent

Written informed consent was obtained from all participants prior to data collection.

## Conflicts of Interest

The authors declare no conflicts of interest.

## Supporting information


**Data S1:** jan70334‐sup‐0001‐DataS1.pdf.

**Data S2:** jan70334‐sup‐0002‐DataS2.pdf.

**Data S3:** jan70334‐sup‐0003‐DataS3.docx.

**Data S4:** jan70334‐sup‐0004‐DataS4.docx.

**Data S5:** jan70334‐sup‐0005‐DataS5.docx.

## Data Availability

The dataset contains sensitive, context‐specific information drawn from a small sample, and even with pseudonymisation, the risk of deductive disclosure remains. As a result, public access to full transcripts is restricted based on ethical considerations. The anonymised excerpts included in the paper are identified only by general codes (e.g., M01, M02, etc.) to preserve participant confidentiality. Requests for access to the underlying data may be submitted to the overseeing ethics body, subject to institutional review and appropriate safeguards.
