# Supplementary material for: Optimising Psychosocial Interventions for Parents Following Perinatal Bereavement: A Qualitative Study of Midwives' Perspectives
Source: J Adv Nurs. 2025 Nov 3;82(7):7413–27. doi: 10.1111/jan.70334 (PMC13267433; doi:10.1111/jan.70334)
Supplement: Supplementary file 2 — Data S2: jan70334‐sup‐0002‐DataS2.pdf. [file JAN-82-7413-s005.pdf]

GRIPP2 short form

Section and topic	Item	Reported on page No
1: Aim	Report the aim of PPI in the study	P 6
2: Methods	Provide a clear description of the methods used for PPI in the study	P 6
3: Study results	Outcomes—Report the results of PPI in the study, including both positive and negative outcomes	P 6
4: Discussion and conclusions	Outcomes—Comment on the extent to which PPI influenced the study overall. Describe positive and negative effects	P 6
5: Reflections/critical perspective	Comment critically on the study, reflecting on the things that went well and those that did not, so others can learn from this experience	P 6

PPI=patient and public involvement
