# Supplementary material for: Optimising Psychosocial Interventions for Parents Following Perinatal Bereavement: A Qualitative Study of Midwives' Perspectives
Source: J Adv Nurs. 2025 Nov 3;82(7):7413–27. doi: 10.1111/jan.70334 (PMC13267433; doi:10.1111/jan.70334)
Supplement: Supplementary file 3 — Data S3: jan70334‐sup‐0003‐DataS3.docx. [file JAN-82-7413-s003.docx]

**File S3.** Interview Prompts of Midwives

**Opening and introduction:**

*Very nice to meet you. Self-introduction. Thank you for participating in our study. We are developing a psychosocial intervention (PSI) that midwives can use when working with parents experiencing perinatal loss (miscarriage, stillbirth, neonatal death, or termination of pregnancy for foetal abnormalities).*

*In this interview, we want to:*

*1. Understand midwives’ experience of current perinatal bereavement care.*

*2. Understand midwives’ perceptions and needs of providing a new midwife-led PSI.*

*3. Identify the barriers and facilitators to provide better care.*

***This Interview should take around an hour.*** *If at any point you want to stop or want any clarification on anything, just let me know. Thank you so much. Before we begin, please let me start recording this interview, but reassure you that your details will be kept confidential.*

*Do you have any questions before we start?*

**Part 1: Experiences of current perinatal bereavement support**

Current Ways of Working:

1. Can you give me a brief introduction about your background and your work regarding perinatal bereavement care?

- At what point do you interact with them?
- What do you do for them?
- How do you interact with them when you do this?
- What areas of care/interaction do you think you do well/not well?
- **Evidence/practice guidelines/Training (study days)?**
- Do you see emotional support as a part of your role? if so, how (evidence)? Identify/screening/assessment?
- **What works well/doesn’t work well / How well does it work?**
- What support information is available for parents to access support in the local communities? (Did you use local resources? Local support groups)

Experience of current care:

1. Could you tell me about any Good/Bad experiences (emotional support) that really stand out to you regarding your interactions with bereaved parents?
   - What made the experiences good/bad for you?
   - What did you learn from your experiences that might improve perinatal bereavement care? (Clinical practice, Personal/emotional)
   - **What is your view of existing PBC?**
   - How might the provision of perinatal bereavement care be improved?

**Part 2: Perceptions and needs of future midwife-led PSIs for bereaved parents**

*As you know, this project is about developing a midwife-led psychosocial intervention (PSI) for perinatal bereavement. PSIs mean any interactions aiming to support bereaved parents to better through the grieving process. Like I said before, it can be small things like naming or holding the baby, greeting, meditation, or encouraging positive behaviours and self-care, etc.*

1. **Are there any PSIs you delivered or observed that you think helped the person in that situation? (standard/evidence/training)**
   - Evidence/Training/Experience?
   - What changes are acceptable do you think would improve the PSI?
2. **In your opinion, what would a perfect midwife-led PSI looks like?**
   - What does midwife-led PSI mean to you?
   - What ingredients would you recommend/suggest should go into this intervention? Why?
   - What would the best way to support them? (format, time, place)
   - What other suggestions do you have for us to develop *pragmatic midwife-led PSIs* for bereaved parents?
3. **What do you think matters most to midwives when delivering this perfect intervention? (Midwives' needs, how to address them)**
   - Training needs: what educational content would help?
   - Support needs: what support would you need to deliver this PSI?
   - What would be the best way to train midwives (online? face to face? Webinar, online module? Mandatory CPD (Continuing professional development)?

Part 3: Barriers and facilitators

1. If we developed this perfect intervention, **what factors would motivate you to use this perfect PSI as part of your care for bereaved parents?** What would **stop** you from using it?
2. Think of a time when you were able to easily interact with a bereaved parent. What **enabled you to do that?**

- **Physical Capabilities**: What things have been put in place to help you interacting with bereaved parents?
- **Psychological capabilities** e.g. how would your mental health state affect your interaction? How do you feel when you work with bereaved parents? (enables, support needs);
- **What else challenges** have you encountered when working with bereaved parents? What things have hindered your interaction with bereaved parents? e.g. training, support, time, staff, paperwork, layout of ward etc.
- Based on what you’ve told me, do you have any thoughts about how to **best change or improve** the way you work with parents?

**Thank you so much, that’s very helpful. And that’s all the questions I have.**

**Is there anything else you would like to add?**

*Thank you so much for your time, we appreciate all of your feedback.*
