# Supplementary material for: Optimising Psychosocial Interventions for Parents Following Perinatal Bereavement: A Qualitative Study of Midwives' Perspectives
Source: J Adv Nurs. 2025 Nov 3;82(7):7413–27. doi: 10.1111/jan.70334 (PMC13267433; doi:10.1111/jan.70334)
Supplement: Supplementary file 4 — Data S4: jan70334‐sup‐0004‐DataS4.docx. [file JAN-82-7413-s001.docx]

**File S4.** Characteristics of participants (n=22)

| **Code** | **Research Site** | **Highest Education** | **Years of Experience** | **Current Practice Area** | **Bereavement Training** | **Confidence in Providing PSI** |
| --- | --- | --- | --- | --- | --- | --- |
| M01 | 1 | Master’s degree | 8 | High Dependency Unit, bereavement | Study days | Completely Confident |
| M02 | 3 | Bachelor’s degree | 22 | Midwifery Student Allocations Liaison Officer | Part of midwifery training & study days | Fairly Confident |
| M03 | 3 | Master’s degree | 24 | Manager/leader | Bereavement and termination of pregnancy study days | Fairly Confident |
| M04 | 1 | Bachelor’s degree | 3 | Labour and birth | No | Fairly Confident |
| M05 | 3 | Bachelor’s degree | 16 | Maternity day assessment unit | Teardrop study day | Fairly Confident |
| M06 | 3 | Master’s degree | 21 | Registered Advanced Midwife Practitioners | Study day and local information sessions | Fairly Confident |
| M07 | 1 | Bachelor’s degree | 11 | Gynaecological unit | Study day | Completely Confident |
| M08 | 2 | Bachelor’s degree | 2 | Antenatal care | Dealing with loss study day | Somewhat Confident |
| M09 | 2 | Master’s degree | 10 | Manager/leader | Study days, bereavement module | Completely Confident |
| M10 | 2 | Bachelor’s degree | 6 | Antenatal care | Bereavement in pregnancy study day | Somewhat Confident |
| M11 | 2 | Bachelor’s degree | 2 | Labour and birth | No | Fairly Confident |
| M12 | 2 | Bachelor’s degree | 9 | Antenatal care | Study days | Fairly Confident |
| M13 | 2 | Bachelor’s degree | 3 | Antenatal care | Study day in bereavement care | Fairly Confident |
| M14 | 1 | Master’s degree | 20 | Labour and birth | Prefer not to answer | Completely Confident |
| M15 | 2 | Master’s degree | 19 | Antenatal care | Study days with hospital, I also help in the bereavement department. | Completely Confident |
| M16 | 2 | Bachelor’s degree | 5 | Antenatal care | Dealing with loss study day | Fairly Confident |
| M17 | 2 | Master’s degree | 11 | Antenatal care | Study days | Fairly Confident |
| M18 | 2 | Bachelor’s degree | 37 | Bereavement support midwife | My degree focused perinatal bereavement counselling and psychotherapy. Study days trainer. | Completely Confident |
| M19 | 3 | Higher Diploma | 33 | Bereavement support midwife | Study day/teardrop workshop | Completely Confident |
| M20 | 3 | Master’s degree | 30 | Midwifery Lecturer | Bereavement workshop in the hospital for staff | Somewhat Confident |
| M21 | 1 | Master’s degree | 7 | Bereavement support midwife | Basic training in midwifery degree | Completely Confident |
| M22 | 1 | Master’s degree | 30 | Maternity day assessment unit | No | Somewhat Confident |
