# Supplementary material for: Optimising Psychosocial Interventions for Parents Following Perinatal Bereavement: A Qualitative Study of Midwives' Perspectives
Source: J Adv Nurs. 2025 Nov 3;82(7):7413–27. doi: 10.1111/jan.70334 (PMC13267433; doi:10.1111/jan.70334)
Supplement: Supplementary file 5 — Data S5: jan70334‐sup‐0005‐DataS5.docx. [file JAN-82-7413-s002.docx]

**File S5.** Theme, sub-theme and quotes

| **Themes** | **Sub-themes** | **Quotes** |
| --- | --- | --- |
| Theme 1: Building Relationships as a foundation for PSI delivery | Communication as a bridge | - *“When the parents are asked about their care…it’s not primarily the physical care they’ll come back and talk about, it will be the communication, it will be the compassion…and making that authentic connection, it’s so important.” (M18)* - *“Just trying to equally support the dad as well because I feel like…sometimes dad may feel a little...bit left to the side.” (M11)* - *"How we communicate is really important...being very mindful what we are saying, because once you’ve said something it's said, and it will resonate in a parent's head." (M20)* - *“Just hearing it in normal language...to make it more kind of personal and not as formal... You want them to feel like they can talk to you kind of like they would talk to a friend or family member.” (M16)* - *“Being realistic and open and honest with the women is very important... There were certain questions she was asking me that I didn’t know the answer to. I acknowledged that with her... but I will find out and I’ll come back to you.” (M03)* - *“[Using] open-ended questions…'how do you feel about holding the baby?' rather than 'do you want to hold the baby?'... your open-ended questions can tell you a lot more than your closed questions.” (M20)* - *“The hardest thing is language barriers... you’re trying to care for a woman and give them bereavement support through a phone with another person, and I don’t know exactly if he’s or her is telling the exact same story.” (M11)* - *“I find that when they’re angry…they’ll question everything…it’s really difficult to communicate with them and try and make them at ease and support them.” (M10)* |
|  | Continuity of care builds deeper connection | - *“The patients that I have met at the time of their diagnosis of their loss, I find that I have more of a connection from the beginning... they tell me that it helps them to speak to me after.” (M18)* - *“I suppose I was with her through the whole journey and really bonded with her. And I got a lovely card actually the other day from her… I saw them from start to finish. It was continuity.” (M12)* - *“If I’m in today and I’m in tomorrow, I’ll take the same lady again to have continuity of care… to build the relationship up so there’s more of a trust and support system there.” (M11)* - *“A very clear handover from the midwife... we can write everything down into the chart, so we are not asking the women the same questions forty times... it's continuity for the midwife but also for the obstetrician or anyone else that is reviewing the notes.” (M20)* - *“She has someone, a name, a number, that’s where the continuity comes back to. But only obviously if they know them… it's just trying to keep the lines of communication open.” (M06)* - *“If it's the weekend or a night or a bank holiday weekend kind of thing… that kind of continuity is lost... sometimes it kind of falls when the bereavement midwife wasn’t on.” (M06)* - *“Bereavement support... should be 7 days a week... when you work in the ward, it's not everyone’s job to know all the information... it does cause anxiety amongst staff, and I think it causes anxiety for families.” (M21)* - *“If you have a clinical midwife specialist answering a bleep 24/7 to come to a patient, my worry about that is would the midwives lose their skills?” (M18)* |
|  | Small gestures, deep impact | - *“Sometimes I even collect them from the front door…just to give them a bit of a sense of relief and security.” (M07)* - *“When you go in then it's very important to introduce yourself... to offer your condolences, to acknowledge from the off what they are going through is really tough and that you are sorry that they are going through it.” (M15)* - *“I think being like that, closed off, abrupt and unapproachable, they would definitely be factors I feel would not work with couples.” (M07)* - *“Being the midwife caring for these patients, being approachable and just letting them know that you’re there to support them through it. That they feel safe when you’re there.” (M07)* - *“She went to theatre, I got her husband a cup of tea and then I chatted with him, and she actually sent me a beautiful card and a little key ring to just say how thankful… the little things that we do can really help.” (M07)* - *“My natural reaction would be to provide some form of comfort, you know hold their hand, give them some tissues, touch their back, if they feel like they need a hug or whatever.” (M22)* - *“We do put up the end-of-life sign... so everyone then like lowers their voices, they speak quieter, we lower the foetal monitors down. It just gets very respectful if that sign is up.” (M07)* |
| Theme 2: PSI as the core element of perinatal bereavement care | Facilitating emotional expression | - *“Having that safe space to talk about these feelings, that they’re so worried about and they’re afraid to share with their family, sometimes just having the safe space with me is all that needs.” (M18)* - *“Most people don’t have mental health problems, most people are just going through grief and loss. So it’s important that we realise that and we normalise the feelings of grief and loss.” (M19)* - *“I suppose one of the things I would have learnt is it's okay to sit in the silence as well. And also that’s the first stages of grief, the silence. And you have to be comfortable rather than filling the silence with rubbish.” (M02)* - *“If you are not acknowledging it (the loss) and you are not validating it (her grief), she feels like oh I can’t even be upset in front of her.” (M12)* - *“Emotional support we provide by being with the woman, by being with her partner, listening to them, holding them in that grief and giving them the space to talk, to express themselves.” (M15)* - *“Sometimes saying nothing is enough… We don’t always have to fill the space of silence… It's a very difficult thing to be comfortable with the silence… I learn it every day from our chaplain, and it's a wonderful skill.” (M21)* - *“Journaling is a way of self-expression… it's something you can come back to in time… we can see how much growth we’ve had since then.” (M17)* - *“Writing a letter to the baby, to go into the coffin with the baby, that’s something that I’ve seen has been helpful a few times.” (M12)* - *“We used to have… a little colouring book or a little something that they could put their thoughts down on the paper and colour and draw or something.” (M11)* - *“Meditation, visualisation of where a happy place could be for them, and box breathing… I find it works amazing… better than saying to someone play music and breathe because that means nothing to them. It's when you find a place that you feel safe, it's more effective.” (M15)* - *“The idea of trying to give them a little bit of peace and quiet in their room and have a little candle lighting and stuff can really help them.” (M01)* - *“A room that could be like a meditation space, like you could have a diffuser and a soft lamp and things like this, like a relaxing space rather than being stuck in four walls for however long.” (M13)* |
|  | Supporting bonding and grief through memory-making | - *“Women, it’s a huge shock, it’s a grieving process, it’s a very uncertain time for them... They may feel they just want to move away from it and just forget about it and not interact with it. So it’s a huge, I suppose onus on us to kind of build on that for the woman. Because we know that down the road the women really value that.” (M01)* - *“Féileacáin boxes…there’s lovely little candles in it, there’s teddy bears that we can give for their children at home... it helps mam and dad go home and explain to their children and it’s keepsake for them as well.” (M11)* - *“Acknowledging the baby as a person and I think that is important for the couple to hear… it's important to embrace the baby. I think that helps build up rapport with the couple.” (M12)* - *“It’s very important I think to recognise features of the baby…that’s their story then for their baby… that’s how you make memories and that’s how they get to know their baby.” (M15)* - *“The memory making is probably one of the biggest things…it makes a connection, and it's also inclusive of the family.” (M21)* - *“Some families would say it was really important that they got time to spend with their baby.” (M01)* - *“We are the gatekeeper to make sure that the parents have the time with the baby.” (M06)* - *“Explaining everything…encouraging them…because it's difficult. ‘Can I touch the baby?’, and they don’t even ask—that’s the thing. So it's up to us to encourage it, that it is okay and make it normal for them.” (M06)* - *“I did also meet women who didn’t want photographs, and I suppose we have to accept that too…letting them know that... just because you said no at ten o’clock doesn’t mean you can’t change your mind at two.” (M02)* - *“It’s just a reassurance that there is no what most people do, it’s what works for you… it’s about what you are able to manage at this time, and that’s just enough.” (M21)* |
|  | Empowering through timely and sensitive informational support | - *“They may not be able to take in all the information at once or they may forget it quite easily because they’re so upset and traumatised.” (M03)* - *“It's about not overloading them with everything at once because they can’t take that in…You’re not rushing them making decisions, you’re giving them time to talk about it, to think about it, for the idea to develop with them.” (M03)* - *“We have to empower…we’re giving the information and encouraging them to make the decisions.” (M03)* - *“Allow the women to know this is what we can do for you. We do feel it will be beneficial, however it's at your discretion and you can choose to do it or not to do it at any time.” (M01)* - *“The communication as well between care givers is really important too, that there’s not different care givers going in giving different information.” (M03)* - *“It’s important to kind of signpost the parents to different therapies and organisations that are there and give them the written information.” (M03)* - *“I usually just find out from the parents where they are and find the helpful things that they already have or help them to notice the important things that they already have” (M18)* - *“I suppose in a busy hospital things go get missed…So for me it's about sharing the communication…it isn’t just us going around, it’s the porters, the catering, the household, you know the electricians, the midwives, nurses, doctors, consultants—it’s everyone’s responsibility.” (M21)* - *“We’ve a lot of leaflets and information and I think the biggest thing is timing when to give them the leaflets and information too because you don’t want to overwhelm them with so many leaflets.” (M11)* |
|  | Involving social support for long-term healing | - *“We can’t get the family to support them but we do encourage the couple to find the one or two people that they feel comfortable that they can express their feelings with.” (M18)* - *“You bend the rules and make sure she has other support with her. Like her partner or her sister or mother…you try and help to bridge that gap because you can’t be everywhere—that’s the reality of it.” (M06)* - *“I think counsellors that are appropriately trained and you know giving resources like maybe books and talk to them about how to explain to the sibling what has happened.” (M03)* - *“The chaplain support works very well…[but] it's not for everybody, some people aren’t religious and obviously we are a multicultural society now.” (M12)* - *“Because you are not alone, while everyone’s situation is unique you are not the only one who has suffered and there might be some support in being like it's not just me.” (M04)* |
| Theme 3: Negotiating PSI delivery in a constrained system | Responsive and individualised PSI delivery | - *“I will go by their cues…it’s the couples lead you basically. It’s not a one shoe fits all, couples will tell you most of the time what they need…if they don’t tell you, if you ask, they will let you know.” (M07)* - *“You go into a room and immediately the tone is there, or the vibe is there… based on how they interact with me, I’ll know what emotional support to give, based on their demeanour.” (M04)* - *“People from different counties react differently to bereavement and loss.” (M22)* - *“The big thing is respecting everyone else has different wishes and everyone’s culture and custom and norms.” (M11)* - *"We can’t force them. All we can do is offer them and encourage them if we feel they will bring long-term benefit and healing." (M02)* - *“You are constantly maybe offering and letting them know that because you said no at ten o’clock in the morning doesn’t mean you can’t change your mind at two o’clock in the afternoon.” (M02)* |
|  | Lack of structure and consistency in PSI delivery | - *“We’ve had a lot of like new staff start in our specific ward. And these staff members would be very, very inexperienced...It’s not fair sometimes that it’s the luck of the draw is these parents had a really experienced person and this person wasn’t.” (M01)* - *“There’s not a huge emphasis on spending time doing, giving the emotional support…it would be great if there was a framework…that would support the midwife as well.” (M03)* - *“They (bereaved parents) really value a structured approach to their care…because they can’t think logically…so we’ll do the two together as you feel you’re able for it.” (M03)* - *“There’s not a huge emphasis on spending time doing, giving the emotional support or doing this. So it would be great if there was a framework designed that you know we could support women with that...that could be built into the midwife’s role, that would be great and that would support the midwife as well.” (M03)* - *"Sometimes I feel like guidelines can be helpful if you’re not familiar with a new process or I think it comes in handy to be able to maybe follow some things step by step when you’re not familiar with it." (M08)* - *“In terms of guiding how our interaction is, there isn’t really a major guide as such. It’s kind of just your own personal, what you feel works.” (M08)* - *“if it becomes more uniform the care that we give that it's not one person doing a lot better treatment than another. That they all get the same care.” (M04)* |
|  | Systemic constraints in supportive PSIs | - *“There’s a huge amount of paperwork…you may not have as much time as you'd like to deal with the emotional side of it.” (M03)* - *“Emotional support for me and this is where midwives I think really struggle. Because you know it's notorious, we are short staffed…we can’t provide that if we don’t have time… It really affects us because that’s when we’ll go home upset, saying there was a woman who was bereaved, and I feel I didn’t spend the time with her.” (M15)* - *“If the partner is staying over, they are on a little chair that turns into a bed…some are standing, some are sitting on the windowsill, some are sitting on the end of the bed. It’s not a comfortable space for them.” (M12)* - *“We had a separate room where it was ensuite… That actually made the experience better for both the parents but also for the staff. Because you know, you felt they were being given a better service.” (M02)* - *“I’ve been on the labour ward where a woman in labour and her baby is stillborn. And next door has a baby crying, and people cheering…her heart is broken in the bed.” (M09)* - *“Purchase a CD and a CD player for the room so that we could block out the noises…background music…to support the lady.” (M02)* - *“Just more resources—but like that’s like asking to win the lotto, isn’t it? But that would make it easier.” (M17)* - *“We need more cuddle cots from Féileacáin…we need more of everything.” (M18)* - *“They don’t want to meet other women that are pregnant… so you let them out the back door… but then… are you trying to hide them?” (M03)* - *“He wanted to carry the coffin out to the car…you want to be sensitive to other patients…if they see someone with a coffin, they are so scared.” (M16)* - *“These families are anxious from the moment they found out their diagnosis…if they are waiting around…That causes an awful lot of anxiety.” (M21)* - *“If we feel it’s beyond our ability or if the bereavement counsellor believes it's beyond her ability she will refer across to perinatal mental health.” (M09)* - *“It's very hard to find what is mental health and what is bereavement…Mental health department won’t see those families because they don’t see bereavement as a mental health” (M21)* - *“Our mental health support team will not at any stage link in or liaise with bereavements… there is a gap.” (M04)* - *“If the normal adaptation to grief and loss was not happening… I would refer them to… counselling or back to their GP or the perinatal mental health team.” (M19, Site 1)* - *“We work closely with the perinatal mental health… if there are red flags… they take the form of counselling sessions.” (M18, Site 2)* - *“The counsellors we have here would be our social workers… they would be the people who offer emotional support.” (M21, Site 3)* |
| Theme 4: Navigating emotional labour and professional growth in delivering PSIs | The emotional labour of PSI delivery | - *“They really value if you’re there emotionally to support them. That can be draining, because sometimes you feel oh gosh, I’ve given so much of myself to that woman.” (M03)* - *“I felt bad after that because I don’t feel I could give her the proper care.” (M04)* - *“It’s not my loss, it’s their loss, so I try not to take that on…because if you take that on you are just going to burn out very fast.” (M04)* - *“It’s okay to show that human side, but at the same time you know it is about them. So I think there’s a balance.” (M17)* - *“It could be triggering to look after bereaved parents…it can impact on your own mental health.” (M12)* - *“It’s like a light switch… you had to switch off your base and change. That was really difficult.” (M02)* - *“Your tone, your pace changed, everything changed. That is really emotionally draining…it’s a real roller coaster of emotions for a midwife.” (M05)* - *“Sometimes the same midwives can be tasked with looking after these women…because I’m doing this all the time and I can’t do anymore.” (M03)* - *“There’s always a little bit of sadness. But…it makes me be a little bit more positive in my other kind of interactions or goings on in the same day.” (M01)* |
|  | Support needed for the supporter | - *“I find I kind of feel drained…emotionally and psychologically…and I kind of feel, well where do I go for support? And there’s no support for the midwives.” (M03)* - *“You can talk to management… but there’s no active support. There’s no support specifically for people who deal with bereavement.” (M04)* - *“If I know someone has had a bereavement at home… I try and allocate them down the other side.” (M09)* - *“Outside of the job nobody can relate to it, but everyone else you work with can relate to it. You don’t even have to say anything, you just start crying…we are very supportive of each other.” (M12)* - *“There is a support number here…but nobody ever rings it. How can you ring and say, ‘I was minding a baby that has died, and I’m upset?’ Like what do they even say?” (M15)* - *“It’s important…that you are not going to overstretch that midwife…looking at realistic workloads and expectations.” (M20)* - *“When she would come to the ward, I remember distinctly her first question to me was ‘how are you doing?’ She was a massive support to the midwives…she was our go-to person.” (M02)* - *"It would be nice if we had a peer group for midwives…we could check in on each other in a safe environment to discuss how that went and how we felt and what worked well and what we could improve." (M03)* - *“If the carer isn’t being looked after, they may not be giving the best care.” (M02)* |
|  | Fostering confidence and preparedness through training | - *“I had to learn by maybe making someone else uncomfortable…but having some training would have made it easier for them.” (M04)* - *“The challenge...is we have a fear that we’ll say something wrong or do something wrong.” (M03)* - *“Some people aren’t very good at going in and just letting someone cry and talk…there could be more emotional support kind of study day.” (M13)* - *“Sometimes you think that the woman has something against you, and it makes you feel like a bad midwife.” (M10)* - *“The parents will pick on our discomfort…we need training on what the baby looks like, what the baby could look like.” (M19)* - *“Some of the complaints we hear from families are… that staff don’t acknowledge their baby… I think that comes from fear and not being comfortable around bereavement and death.” (M21)* - *“What enabled me is my experience and the years that I’ve been working in the field… it’s the continuum of experience.” (M22)* - *“It’s role modelling…I think this person did it really well. I’m going to take that from her.” (M20)* - *“None of us were trained with counselling skills but effectively you become a counsellor to the best of your ability.” (M02)* - *“It’s important that all midwives are skilled in the interventions…and it’s not just left to one or two or three midwives on the floor.” (M03)* - *“The more resources we have in our toolbox to be able to respond to parents’ needs…the better we will be able to do our job, and minimise the traumatic effect for the parents.” (M03)* |
